# Imaging protein aggregates in the serum and cerebrospinal fluid in Parkinson’s disease

**DOI:** 10.1093/brain/awab306

**Published:** 2021-08-19

**Authors:** Evgeniia Lobanova, Daniel Whiten, Francesco S Ruggeri, Christopher G Taylor, Antonina Kouli, Zengjie Xia, Derya Emin, Yu P Zhang, Jeff Y L Lam, Caroline H Williams-Gray, David Klenerman

**Affiliations:** 1 Department of Chemistry, University of Cambridge, Lensfield Road, Cambridge CB2 1EW, UK; 2 Laboratory of Organic Chemistry, Wageningen University, Stippeneng 4, Wageningen 6703 WE, The Netherlands; 3 Laboratory of Physical Chemistry, Wageningen University, Stippeneng 4, Wageningen 6703 WE, The Netherlands; 4 Department of Clinical Neurosciences, University of Cambridge, Cambridge CB2 0PY, UK; 5 UK Dementia Research Institute at Cambridge, Cambridge CB2 0XY, UK

**Keywords:** Parkinson’s disease, α-synuclein aggregates, amyloid-β, aggregates, super-resolution imaging, early-disease serum biomarkers

## Abstract

Aggregation of α-synuclein plays a key role in the development of Parkinson’s disease. Soluble aggregates are present not only within human brain but also the CSF and blood. Characterizing the aggregates present in these biofluids may provide insights into disease mechanisms and also have potential for aiding diagnosis.

We used two optical single-molecule imaging methods called aptamer DNA-PAINT and single-aggregate confocal fluorescence, together with high-resolution atomic force microscopy for specific detection and characterization of individual aggregates with intermolecular β-sheet structure, present in the CSF and serum of 15 early stage Parkinson’s disease patients compared to 10 healthy age-matched controls.

We found aggregates ranging in size from 20 nm to 200 nm, in both CSF and serum. There was a difference in aggregate size distribution between Parkinson’s disease and control groups with a significantly increased number of larger aggregates (longer than 150 nm) in the serum of patients with Parkinson’s disease. To determine the chemical composition of the aggregates, we performed aptamer DNA-PAINT on serum following α-synuclein and amyloid-β immunodepletion in an independent cohort of 11 patients with early stage Parkinson’s disease and 10 control subjects. β-Sheet aggregates in the serum of Parkinson’s disease patients were found to consist of, on average, 50% α-synuclein and 50% amyloid-β in contrast to 30% α-synuclein and 70% amyloid-β in control serum [the differences in the proportion of these aggregates were statistically significant between diseased and control groups (*P* = 1.7 × 10^−5^ for each species)]. The ratio of the number of β-sheet α-synuclein aggregates to β-sheet amyloid-β aggregates in serum extracted using our super-resolution method discriminated Parkinson’s disease cases from controls with an accuracy of 98.2% (AUC = 98.2%, *P* = 4.3 × 10^−5^).

Our data suggest that studying the protein aggregates present in serum can provide information about the disruption of protein homeostasis occurring in Parkinson’s disease and warrants further investigation as a potential biomarker of disease.

## Teaser

Using a suite of new biophysical methods, Lobanova *et al*. reveal changes in the composition and size of individual α-synuclein and amyloid-β aggregates in the serum and CSF of patients with Parkinson's disease compared to controls, with a higher proportion of larger aggregates and an increased α-synuclein/amyloid-β ratio developing in disease.

## Introduction

Protein aggregation, due to disrupted protein homeostasis, plays a central role both in ageing and in the pathogenesis of many debilitating neurodegenerative disorders such as Alzheimer’s and Parkinson’s diseases. In Alzheimer’s disease and Parkinson’s disease, there is accumulation of aberrantly processed and misfolded proteins such as amyloid-β,^1^ tau^2^ and α-synuclein (α-syn).^3^ These proteins lose their physiological roles, aggregate, and acquire novel neurotoxic functions, leading to the accumulation and spread of aggregates.^4^ Protein aggregates are removed by a wide number of mechanisms, both intracellularly and extracellularly.^5^ Over time, these clearance systems become unable to cope with the excessive neurotoxic burden leading to slow accumulation of intracellular aggregates.^6^ This disrupted protein homeostasis contributes to progressive neuronal death by a variety of different mechanisms that are believed to be initiated by soluble protein aggregates,^6-8^ and the disruption is thought to be due to a decreased rate of aggregate removal.^9^

A major route of aggregate removal from neurons is secretion into the interstitial fluid and removal by phagocytosis and endocytosis by glial cells, but aggregates also reach the CSF via the glymphatic system, and efflux into the peripheral blood and lymphatic system, where they are removed by macrophages and tissues such as the liver.^10^ The presence of secreted forms of α-syn has been reported in mouse and human brains showing significant levels of α-syn in the interstitial fluid of both Parkinson’s disease and non-Parkinson’s disease cases.^11^ In particular, total α-syn concentration in the interstitial fluid of the A53T transgenic mice was 0.5 ng/ml compared to 0.15 ng/ml in control mice. Interstitial fluid in human (non-Parkinson’s disease) brain contained 0.5–8 ng/ml of total α-syn with comparable levels in CSF. In contrast, CSF concentrations of α-syn oligomers were reported at 50–100 pg/ml^12^ in healthy individuals with an approximate 1.5-fold increase in Parkinson’s disease patients, based on sensitive ELISA measurements. Levels of amyloid-β in CSF are considerably lower: Alzheimer’s disease CSF contains only 1–2 pg/ml of amyloid-β oligomers^13^ compared to 0.4–0.7 pg/ml in aged control CSF. This is likely to be due to the fact that most of the amyloid-β aggregates are deposited into inert insoluble amyloid-β plaques in the brain. However, 40% of all amyloid-β produced in the brain is removed in the periphery.^14^ Measurements of the rate of amyloid-β deposition in the brain by Masters and colleagues^15^ show that in normal brain 2 mg of amyloid-β is deposited in 19 years compared to 6 mg in an Alzheimer’s disease brain, which is a surprisingly small difference in deposition by a factor of 3 over an extended time period. Thus, the existing data suggest that a major route of monomer and aggregate removal is secretion by a variety of mechanisms into the interstitial fluid, where they are either deposited in insoluble aggregates (in the case of amyloid-β predominantly) or enter the CSF for removal.

Surprisingly, the characteristics of the soluble oligomeric species that are present in human brain and biofluids have not been clearly established, nor which species are most neurotoxic and contribute to disease onset and progression. We have, to date, lacked the tools to effectively measure and quantify these oligomeric species due to their low concentration down to subpicomolar scale, and heterogeneity in both size and structure. However, characterization of the aggregates present in both CSF and serum provides an opportunity to follow the changes in protein aggregation during the development of neurodegenerative disease. To date, the main method used to detect protein aggregates in serum and CSF has been based on antibody capture, using ELISAs. These measurements provide information about the total mass of soluble aggregates present but cannot distinguish between a small number of large aggregates or a large number of small aggregates. Furthermore, in most studies only aggregates of one protein are measured rather than measuring both the amyloid-β and α-syn aggregates present in the same sample. In contrast, we have recently developed a super resolution-based method to measure the number and size of both amyloid-β and α-syn β-sheet protein aggregates in CSF.^16^ Using this method, we found that there was no change in the aggregate number in CSF during the development of Alzheimer’s disease but a change in the length distribution. In combination with high-resolution atomic force microscopy (AFM), our methods showed that a higher proportion of longer aggregates were present in the CSF of Alzheimer’s disease patients leading to an increased inflammatory response *in vitro*. Such an approach of measuring aggregates of multiple protein types may also be of particular relevance in Parkinson’s disease, given that co-pathologies are known to occur in this condition, with a significant proportion of patients having Alzheimer’s type pathology within the brain.^17^ We have therefore used our quantitative super-resolution imaging methods combined with high-resolution imaging of aggregate morphology and immunodepletion to detect and characterize the aggregates present in the CSF and serum of Parkinson’s disease patients, compared to similarly-aged control subjects.

## Materials and methods

### Participants

Idiopathic Parkinson’s disease patients fulfilling UK Parkinson’s disease Brain Bank Criteria and within 2 years of diagnosis were recruited from the Parkinson’s Disease Research Clinic at the John Van Geest Centre for Brain Repair, Cambridge, UK. Age and sex-matched healthy control volunteers were recruited from the NIHR Cambridge Bioresource (http://www.cambridgebioresource.org.uk) or were healthy spouses or carers of patients attending our Research Clinic. Demographic data were collected and all participants completed neuropsychological testing including the Addenbrooke’s Cognitive Examination version 3 (ACE-III). Participants with Parkinson’s disease were also assessed with the Movement Disorder Society Unified Parkinson’s Disease Rating Scale (MDS-UPDRS) and disease stage was determined using the Hoehn and Yahr scale (see Tables 1 and 2 for details). Ethical approval was obtained from the East of England—Essex Research Ethics Committee (16/EE/0445) and informed consent was obtained from all the participants.

**Table 1:** Demographic and clinical characteristics of Parkinson’s disease and control participants included in confocal imaging and AD-PAINT experiments

	Serum			CSF		
	Control	Parkinson’s disease	*P*-value	Control	Parkinson’s disease	*P*-value
Sample size	10	15	0.4	10	15	0.4
Age, years	67.8 ± 9.2	67.8 ± 7.9	>0.99	69.3 ± 7.9	67.8 ± 7.6	0.6
Sex, % male	70%	67%	1.0	60%	60%	1.0
ACE-III	91.7 ± 10.8	91.2 ± 3.9	0.9	88.8 ± 12.7	92.5 ± 4.0	0.3
Disease duration, years	–	1.3 ± 0.7	–	–	1.1 ± 0.7	–
Hoehn and Yahr	–	1.8 ± 0.4	–	–	1.7 ± 0.5	–
MDS-UPDRS III	–	27.5 ± 10.6	–	–	27.7 ± 8.9	–
MDS-UPDRS Total	–	46.8 ± 15.1	–	–	46.9 ± 14.1	–

Values represent the mean ± SD. Variables were compared using the permutation (exact) test except the sample size for which the binomial test was used (*P < 0.05). Control = healthy control.

**Table 2:** Demographic and clinical characteristics of the Parkinson's disease and control participants included in the immunodepletion experiment

	Cohort 1			Cohort 2			Combined		
	Control	Parkinson’s disease	*P*-value	Control	Parkinson’s disease	*P*-value	Control	Parkinson’s disease	*P-*value
Sample size	5	5	1	5	6	1	10	11	1
Age, years	62.4 ± 10.5	64.2 ± 9.2	0.8	65.3 ± 8.0	70.2 ± 10.1	0.4	63.8 ± 8.9	67.5 ± 9.7	0.4
Sex, % male	60%	40%	1	20.0%	83.3%	0.1	40.0%	63.6%	0.4
ACE-III	99.0	94.5 ± 4.0	0.5	97.3 ± 0.6	92.7 ± 4.5	0.1	97.8 ± 1.0	93.5 ± 4.2	0.1
Disease duration, years	–	0.5 ± 0.1	–	–	2.2 ± 2.2	–	–	1.4 ± 1.8	–
Hoehn and Yahr	–	1.6 ± 0.9	–	–	1.8 ± 0.4	–	–	1.7 ± 0.6	–
MDS-UPDRS III	–	30.0 ± 19.1	–	–	30.6 ± 12.9	–	–	30.3 ± 15.4	–
MDS-UPDRS Total	–	54.6 ± 31.7	–	–	52.2 ± 15.9	–	–	53.4 ± 23.6	–
Sample storage duration, years		1.56 ± 0.02			0.32 ± 0.05			1.55 ± 0.42	

Values represent the mean ± SD except the sample storage duration for which the median ± median absolute deviation values were used due to the nonparametric statistics of the data. Variables were compared using the permutation (exact) test except the sample size for which the binomial test was used (*P < 0.05).

### Serum and CSF sampling

Venous blood samples were collected in 7.5 ml S-Monovette tubes from 26 Parkinson’s disease patients and 20 healthy control subjects. After collection, the samples were left to clot at room temperature for 15 min and then centrifuged at 2000 rpm for 15 min. The supernatant (serum) was collected and stored at −80°C until use. Lumbar puncture was performed with 1% lignocaine local anaesthetic in 15 Parkinson’s disease patients and 10 control subjects. CSF (5 ml) was collected and centrifuged for 10 min at 300*g* at 4°C. The supernatant was collected and stored at −80°C. Serum and CSF samples were matched and collected at the same time for 10 of the Parkinson’s disease patients and six control subjects. The exact number of samples (*n*) included in each experiment together with participant demographics are specified in Tables 1 and 2.

### Single-molecule confocal measurements

Aliquots of CSF or serum were diluted by a factor of 2 or 40, respectively, in PBS buffer with pFTAA dye (30 nM). Diluted samples were withdrawn through a single-channel microfluidic device (width = 100 µm, height = 25 µm, length = 1 cm), at a flow velocity of 0.56 cm/s to a syringe (1 ml, HSW, Normject) via polyethylene tubing (0.38 mm ID, Intramedic). Flow control was achieved with syringe pumps (Harvard apparatus PhD Ultra), and the device placed on the microscope stage. Single-molecule confocal experiments were performed on the instrument as described previously.^18^ In brief, a 488 nm laser beam (Cyan CDRH diode laser, Spectra-Physics) was directed to the back aperture of an inverted microscope (Nikon Eclipse TE2000-U). The beam was reflected by a dichroic mirror (FF500/646-Di01, Semrock) and focused to a concentric diffraction-limited spot, 10 μm into the sample in a microfluidic detection channel through a high numerical aperture oil-immersion objective (Apochromat 60×, NA 1.40, Nikon). Fluorescence was collected with the same objective, passing through the dichroic mirror, and imaged onto a 50 μm pinhole (Melles Griot) to remove out-of-focus light. The emission was filtered (510ALP and 535AF45, Omega Optical Filters) and directed to an avalanche photodiode (APD, SPCM-14, PerkinElmer Optoelectronics). A custom-programmed field-programmable gate array (FPGA, Colexica), was used to count the signals from the APD and combine these into time bins, which were selected according to the expected residence time of molecules passing through the confocal probe volume. At each time point data were collected for 10 min (3 × 10^6^ time bins, bin-width 0.2 ms).

### Analysis of single-molecule confocal measurements

The experimental output data were collected using an FPGA card and analysed in Python using custom-written code. To compensate for the variable noise within and between samples the background was first calculated as the rolling average of 3000 time bins. With an approximate event frequency of 1/3000 bins, the presence of even bright events had a negligible effect on the background value calculated. To count the number of events in each sample, the threshold was set at double the background plus 20 AFU. This formula was applied equally to each sample.

### Atomic force microscopy imaging

Samples were diluted 100-fold in PBS buffer and imaged on freshly cleaved mica substrates using AFM. Diluted samples (10 μl) were deposited on the substrate at room temperature. The samples were incubated for 10 min followed by rinsing with 1 ml milli-Q water, then dried using a gentle flow of nitrogen gas.

AFM maps of 3D morphology of all the samples were acquired in a regime of constant phase change, with 2–4 nm/pixel resolution using a NX10 (Park Systems) operating in non-contact mode.^19^ This set-up was equipped with a silicon tip with a nominal radius of <10 nm and spring constant of 5 N/m (PPP-NCHR).

Scanning probe image processor (SPIP) (Image Metrology, Denmark) software was used for image flattening and single aggregate statistical analysis (*n* > 150). The average level of noise for each image was measured using SPIP software, which was in the order of 0.1 nm.^20^ All the measurements were performed at room temperature.

### AD-PAINT imaging

Aptamer DNA-PAINT (AD-PAINT) was performed as previously described.^21^ In brief, round glass coverslips were cleaned using an argon plasma cleaner for at least 1 h. A multi-well chambered coverglass (CultureWell CWCS-50R-1.0) was then attached on the top of the coverslip. The wells were cleaned with the coating buffer (PBS/1% Tween-20) for 45 min followed by an additional incubation with fiducial markers (1:8000, TetraSpeck™ beads, 0.1 µm diameter, Thermo Fisher), diluted in the coating buffer for 15 min. The respective biofluid (10 µl) (CSF or neat serum) or supernatant from the immunodepletion experiment (diluted or neat as specified below) were added to each well and incubated for 30 min to allow any aggregates contained in the fluid to stick to the Tween-20-coated glass surface. In the immunodepletion experiments, the serum in Cohort 2 (Table 2) had a significantly higher concentration of aggregates compared to the samples in Cohort 1 (Table 2) and was therefore diluted 10-fold in PBS prior to adding to the glass surface to work in the quantitative range of AD-PAINT. To control for any non-specific binding to the glass surface, the same volume of PBS buffer was used as a negative control following the same procedure. The wells were then washed three times with PBS and 5 μl of an imaging mixture solution consisting of a 2 nM imaging strand (sequence CCAGATGTAT-CY3B) and 100 nM aptamer-docking strand (sequence GCCTGTGGTGTTGGGGCGGGTGCGTT-ATACATCTA) in PBS was pipetted into each well. To prevent sample evaporation, the wells were sealed with a clean coverslip. All buffers were passed through a 0.02 μm filter (Anotop25, Whatman, Cat. 516–1501) before use. Imaging was carried out using a home-built total internal reflection fluorescence (TIRF) microscope based on a Ti-E Eclipse inverted microscope (Nikon) equipped with a 100× 1.49 NA oil-immersion objective (UPLSAPO, 100×, TIRF, Olympus) and a perfect focus system. The Cy3B was excited with a 561 nm laser source (Cobalt Jive, Cobalt), filtered with a single-band exciter (FF01-561/14–25, Semrock) transmitting 554–568 nm and coupled into the sample by a dichroic mirror (Di01-R405/488/561/635, Semrock) at 561 nm. The fluorescence was separated from the excitation light using the same dichroic (Di01-R405/488/561/635, Semrock), filtered by a long-pass emitter (LP02-568RS-25, Semrock) transmitting 581.3–1281.7 nm and imaged onto the air-cooled EMCCD camera (Photometrics Evolve, EVO-512-M-FW-16-AC-110) operating in frame transfer mode (electron-multiplying Gain of 11.5 e-1/ADU and 250 ADU/photon). Six thousand frames were acquired with an exposure time of 50 ms using a 100× 1.49 NA TIRF objective as described above and a 1.5× tube lens. To minimize any bias associated with the region of interest selection, an automated script (Micro-Manager) was used to collect images in a grid. Images were analysed using the PeakFit ImageJ plugin of the GDSC Single Molecule Light Microscopy package and custom scripts written in Python. The data analysis is described in detail in Whiten *et al*.^21^

### Immunodepletion

Immunodepletion of α-syn and amyloid-β proteins from serum samples (*n* = 11 Parkinson’s disease and 10 control subjects, assayed in duplicate) was performed using the following immunoprecipitation protocol: 5 μg of purified mouse anti-α-syn antibody (1:10, 250 μg/ml, 15–123, SYN-1, BD Transduction Laboratories) or purified mouse anti-amyloid-β antibody (1:40, 1 mg/ml, 1–16, 6E10, BioLegend) or mouse IgG1 isotype control antibody (1:40, 1 μg/ml, COLIS69A, Kingfisher Biotech) (a negative control) were incubated with 1.5 mg magnetic Dynabeads^®^ protein G (30 mg/ml, Invitrogen) in a total volume of 200 μl 0.02% Tween-20 (PBST) in separate 1.5 ml Protein LoBind Tubes (Eppendorf AG) on a rotating wheel at 4°C for 2 h. After spinning down the immunoprecipitated antibody-bead complexes, the Eppendorf tubes were placed on a magnetic rack and the supernatants were aspirated. The beads were then washed twice with 500 μl PBST to remove non-specific binding. The respective serum (200 μl) was added to each Eppendorf tube and rotational incubation was continued overnight at 4°C. For specificity control immunoprecipitation experiments, α-syn or amyloid-β_1__-40_ aggregates at 500 nM or a mixture of α-syn and amyloid-β_1__-40_ aggregates at 500 nM and 2 μM concentrations for both were incubated with the antibody-bead complexes in parallel. After the incubation and centrifugation steps, the supernatants were collected, and 40 µl aliquots were transferred into fresh 0.5 ml Protein LoBind Tubes (Eppendorf AG). The collected aliquots were kept in the freezer at −80°C until further evaluation with AD-PAINT.

### Preparation of α-synuclein and amyloid-β aggregates

Monomeric α-syn was expressed and purified in *Escherichiacoli* according to Hoyer *et al*.^22^ For the aggregation reaction, the stock was diluted in prefiltered PBS (pH 7.4, 0.02 µm Whatman, 6780–1302 filters) to a concentration of 70 µM and incubated at 37°C under constant shaking (200 rpm) for 3 days. Amyloid-β_1-40_ (AS-24235) was purified according to the suppliers protocol. Monomeric amyloid-β_1__-40_ was diluted in prefiltered PBS (pH 8.0) to a final concentration of 20 µM and incubated at 37°C for 2 days. Afterwards, the aggregates were kept at 4°C prior to use for a maximum of 1 week.

### Statistical analysis

All data were analysed using MATLAB (R2020b). Statistical differences for each variable of interest between the Parkinson’s disease and control groups were assessed by the permutation (exact) test, except for the aggregate length and the sample size, for which the Wilcoxon rank sum test for medians and the binomial test were used, respectively. Statistical signiﬁcance was indicated when *P* < 0.05. Data are shown as mean ± standard deviation (SD) with each dot representing individual participants. The lower and upper boundaries of the box indicate the 25th and 75th percentiles, respectively. The relationship between the number of aggregates as well as the mean length of aggregates in serum versus CSF was assessed using the Pearson’s correlation analysis for the serum-CSF matched samples. The correlation (*R*) was considered to be statistically significant when *P* < 0.05. The statistical difference in aggregate numbers between Parkinson’s disease patients and healthy controls in matched CSF and serum was examined by fitting a linear regression model

(1) $$\left(y∼{b_{0}}^{\mathrm{Parkinson}`s\;\mathrm{disease}}+{b_{1}}^{\mathrm{Parkinson}`s\;\mathrm{disease}}\cdot x\;\mathrm{and}\;y∼b_{0}^{\mathrm{HC}}+b_{1}^{\mathrm{HC}}\cdot x\right)$$

with individual coefficients for Parkinson’s disease and healthy control groups and testing the respective coefficients are the same (b0Parkinson's disease= b0HC and b1Parkinson's disease= b1HC). Prediction of disease status by relative β-sheet α-syn content, the β-sheet α-syn/amyloid-β ratio and total number of aggregates was examined by receiver operating characteristic (ROC) analysis. Accuracy of each tested biomarker was evaluated by area under the curve (AUC) whereas the optimal sensitivity and specificity were calculated according to Hajian-Tilaki.^23^ The optimal threshold values for relative α-syn content and the β-sheet α-syn/amyloid-β ratio were assessed using logistic regression.

### Data availability

The data generated in this study are available from the corresponding author upon reasonable request.

## Results

**Figure 1:**
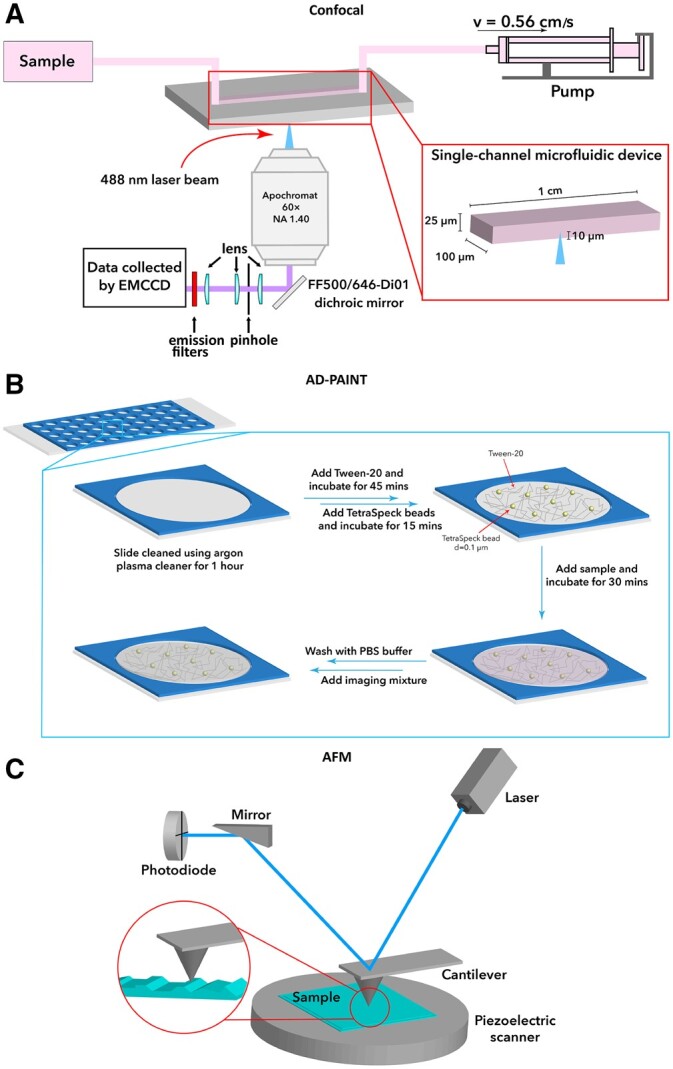
**Schematic of single aggregate methods used.** (**A**) Confocal imaging, (**B**) AD-PAINT and (**C**) AFM.

**Figure 2:**
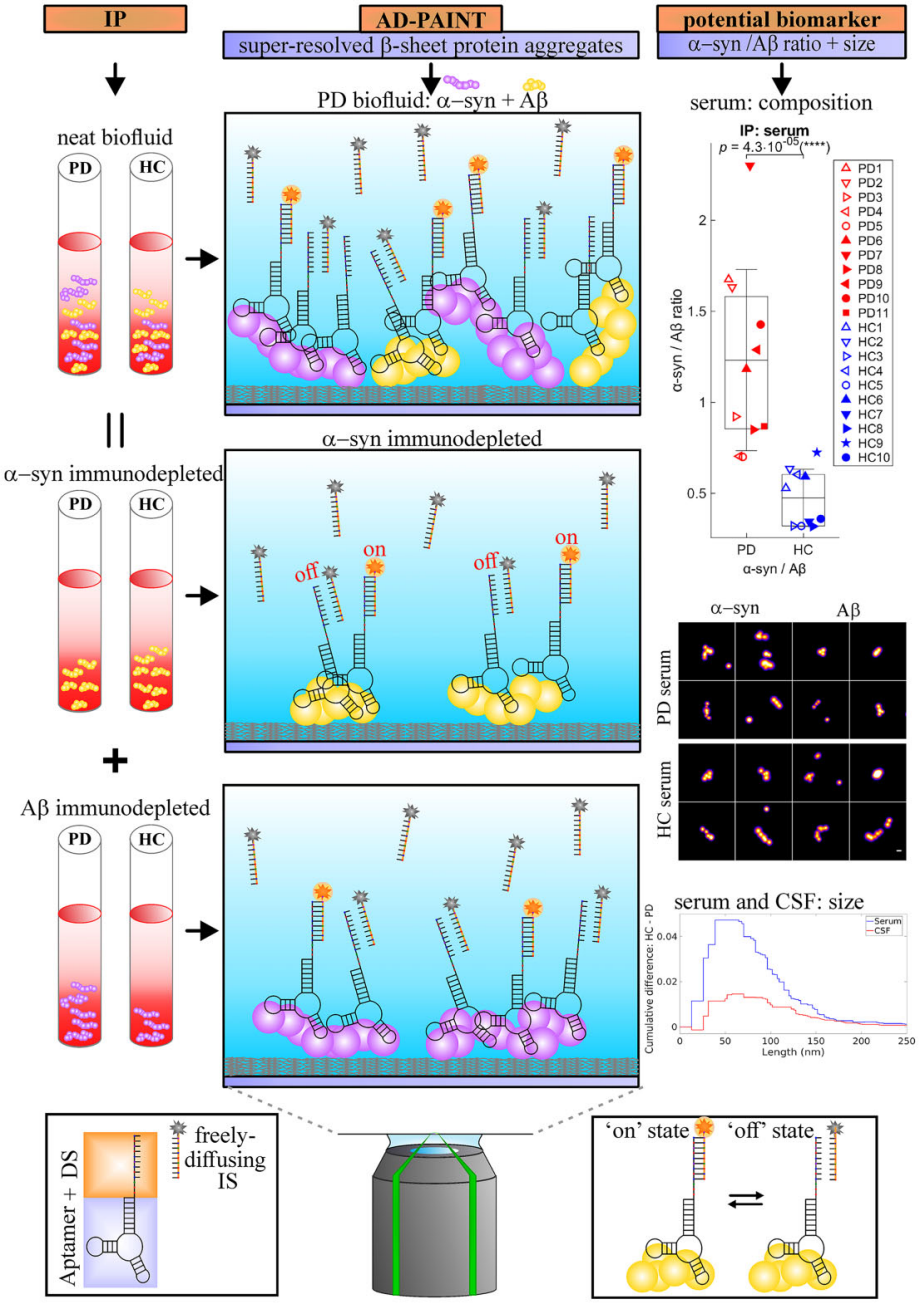
**Overview of experimental design combining AD-PAINT with immunoprecipitation assay for determining the chemical composition of protein aggregates in human biofluids (serum, CSF, etc).** Schematic illustration showing the principle of the AD-PAINT method is also shown. Aβ = amyloid-β; DS = docking strand; HC = healthy control; IP = immunoprecipitation; IS = imaging strand; PD = Parkinson’s disease.

**Figure 3:**
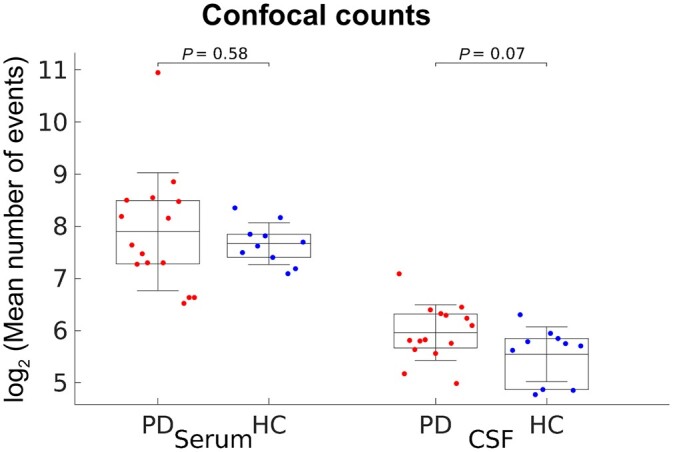
**Confocal analysis of the aggregates present in Parkinson’s disease serum and CSF (*n* = 15) compared to controls (*n* = 10).** For 10 Parkinson’s disease and six healthy control participants, paired serum and CSF samples were collected from the same individuals. Serum was diluted by a factor of 40 and CSF by a factor of 2. Data are shown as mean ± SD with each dot representing individual participants and plotted in log_2_ scale. The lower and upper boundaries of the box indicate the 25th and 75th percentiles, respectively. Parkinson’s disease versus control comparisons using the permutation (exact) test were insignificant (*P >* 0.05). HC = healthy control; PD = Parkinson’s disease.

We used a variety of methods sensitive to single aggregates to detect and characterize the aggregates present in CSF and serum from Parkinson’s disease and healthy controls, as shown in Figs 1 and 2. A summary of study participants is given in Table 1. The dye PFTAA was used to detect aggregates containing β-sheet structures as they flow through a confocal volume.^18^ These experiments show that β-sheet structured aggregates are present in both CSF and serum. The serum was diluted 40-fold and the CSF was diluted 2-fold and a comparable number of counts were detected, indicating about 20-fold more PFTAA active aggregates present in the serum than the CSF. We found no significant difference in the number of PFTAA active aggregates between Parkinson’s disease patients and controls for both CSF and serum (Fig. 3). Our findings were similar in the matched serum and CSF samples (*n* = 10 for Parkinson’s disease and *n* = 6 for healthy controls, *P* > 0.05; Supplementary Fig. 1A). ROC analysis for prediction of disease status gave AUC of 54% for serum aggregates and 71% for CSF aggregates (Supplementary Fig. 1C). To assess the relationship between number of aggregates in serum and CSF, we performed Pearson’s correlation analysis on the patient matched samples and observed no significant correlation between serum and CSF aggregates’ levels in both Parkinson’s disease (*R* = 0.43, *P* = 0.22) and control (*R* = 0.34, *P* = 0.51) subjects (Supplementary Fig. 1B).

**Figure 4:**
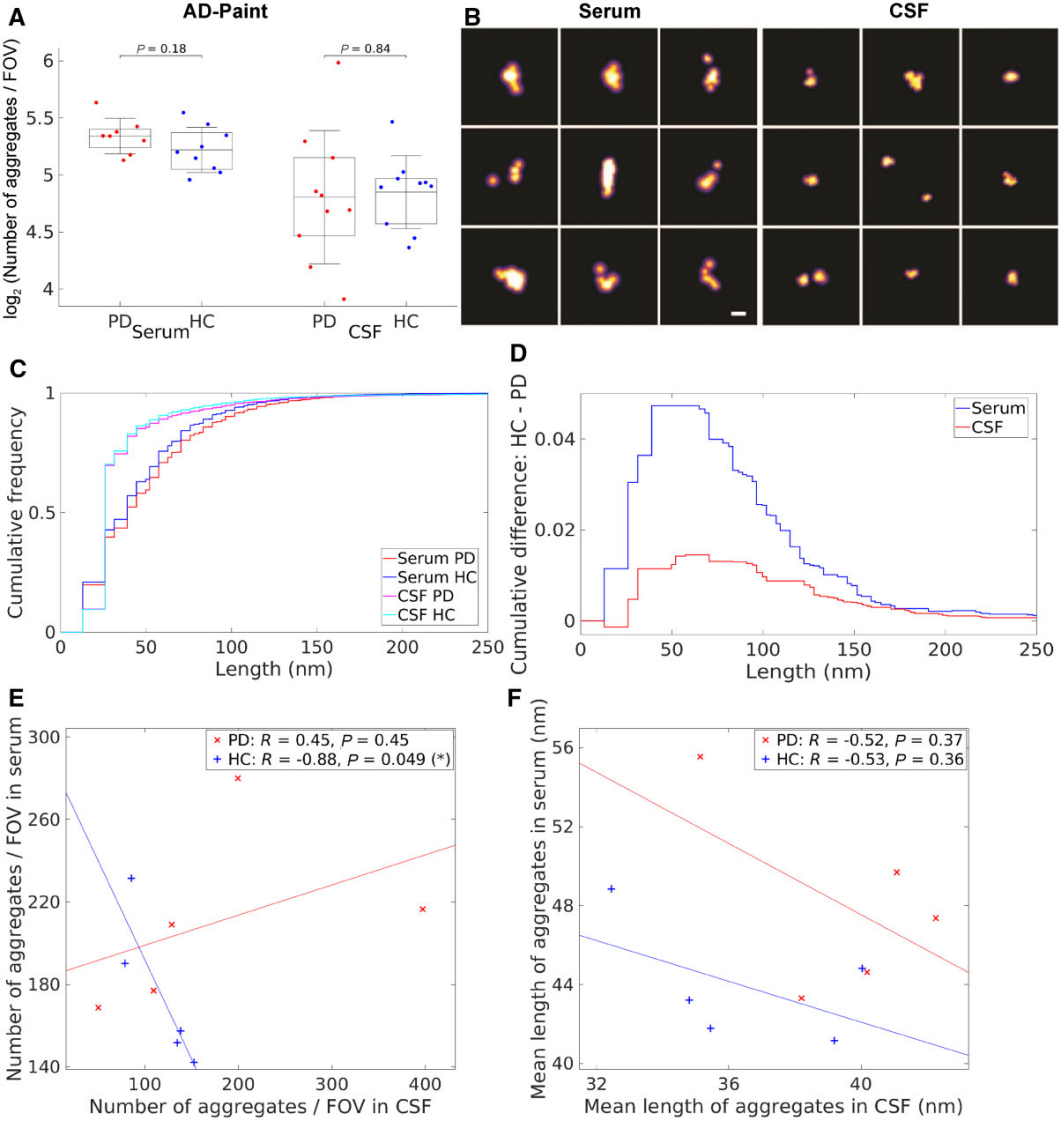
**Characterization of the protein aggregates detected in Parkinson’s disease (*n* = 8) compared to healthy control serum (*n* = 9) and Parkinson’s disease (*n* = 10) compared to healthy control CSF (*n* = 10) using AD-PAINT.** (**A**) Quantification of the number of the aggregates detected by AD-PAINT. For five Parkinson’s disease (PD) and five healthy controls (HC), serum and CSF samples were paired. Parkinson’s disease versus control comparisons using the permutation (exact) test were insignificant (*P >* 0.05). Serum aggregates were undiluted and CSF was diluted 2-fold. (**B**) Examples of super-resolution images taken using AD-PAINT for Parkinson’s disease CSF and serum samples. Scale bar = 100 nm. (**C**) Cumulative length distributions for Parkinson’s disease and healthy control serum and CSF samples measured by AD-PAINT. (**D**) Difference between Parkinson’s disease and control cumulative length distributions for CSF and serum retrieved from **C**. (**E**) Correlation between the number of aggregates in serum versus CSF for matched Parkinson’s disease (red) and healthy controls (blue) subjects. (**F**) Correlation between mean length of aggregates in serum versus CSF for matched Parkinson’s disease (red) and control (blue) samples. Pearson’s correlation coefficients (*R*) and *P*-values are indicated. FOV = field of view.

We then used AD-PAINT to image the individual aggregates in serum and CSF, adsorbed onto a glass coverslip, and measure their size with 20 nm spatial resolution.^21^ This method uses an aptamer that binds to fibrillar aggregates of α-syn and amyloid-β (Fig. 2). Representative images are shown in Fig. 4B. Analysis of the results showed no difference in the number of aggregates detected in both serum and CSF in Parkinson’s disease versus controls (Fig. 4A). Findings were similar in the cohort with matched serum and CSF (*n* = 5 for both Parkinson’s disease and healthy control groups, *P* > 0.05; Supplementary Fig. 2A). We found a strong and statistically significant negative correlation between the number of aggregates in serum versus CSF for matched control subjects (*R* = −0.88, *P* = 0.049; Fig. 4E). In contrast, the aggregates’ levels in Parkinson’s disease serum tended to positively correlate with that in matched Parkinson’s disease CSF but this correlation was not statistically significant (*R* = 0.45, *P* = 0.45; Fig. 4E). However, aggregate numbers between Parkinson’s disease patients and healthy controls in matched CSF and serum were found not to be significantly different (*P* = 0.19) using linear regression analysis (b0Parkinson's disease=184, b1Parkinson's disease=0.15, $b_{0}^{\mathrm{HC}}=288$, $b_{1}^{\mathrm{HC}}=-0.96$) (refer to the ‘Statistical analysis’ section for details). ROC analysis for prediction of disease status gave AUC of 67% for serum aggregates and 58% for CSF aggregates (Supplementary Fig. 2B).

We also analysed the cumulative size distributions of the aggregates found within the CSF and serum samples (Fig. 4C and D). In both biofluids, aggregates were highly heterogeneous in size, ranging from 20 nm, our limit of resolution, to 250 nm. There was a small difference in cumulative size distributions between Parkinson’s disease and control CSF of ∼1%, with Parkinson’s disease samples having a higher proportion of longer aggregates, but this was not statistically significant (*P* = 0.6). There was a greater difference in aggregate size between Parkinson’s disease and control serum of 5%, with Parkinson’s disease serum containing a significantly higher proportion of aggregates longer than 150 nm (*P* = 0.03). In the matched samples, this cumulative difference was ∼2.5% and 5% for CSF and serum, respectively (Supplementary Fig. 2D). These results show that the aptamer used in AD-PAINT detects no change in the total number of aggregates, but a higher proportion of longer aggregates in Parkinson’s disease compared to control serum. We found no statistically significant correlation between mean length of aggregates in matched serum and CSF for both Parkinson’s disease (*R* = −0.52, *P* = 0.37) and control (*R* = −0.53, *P* = 0.36) groups (Fig. 4F).

**Figure 5:**
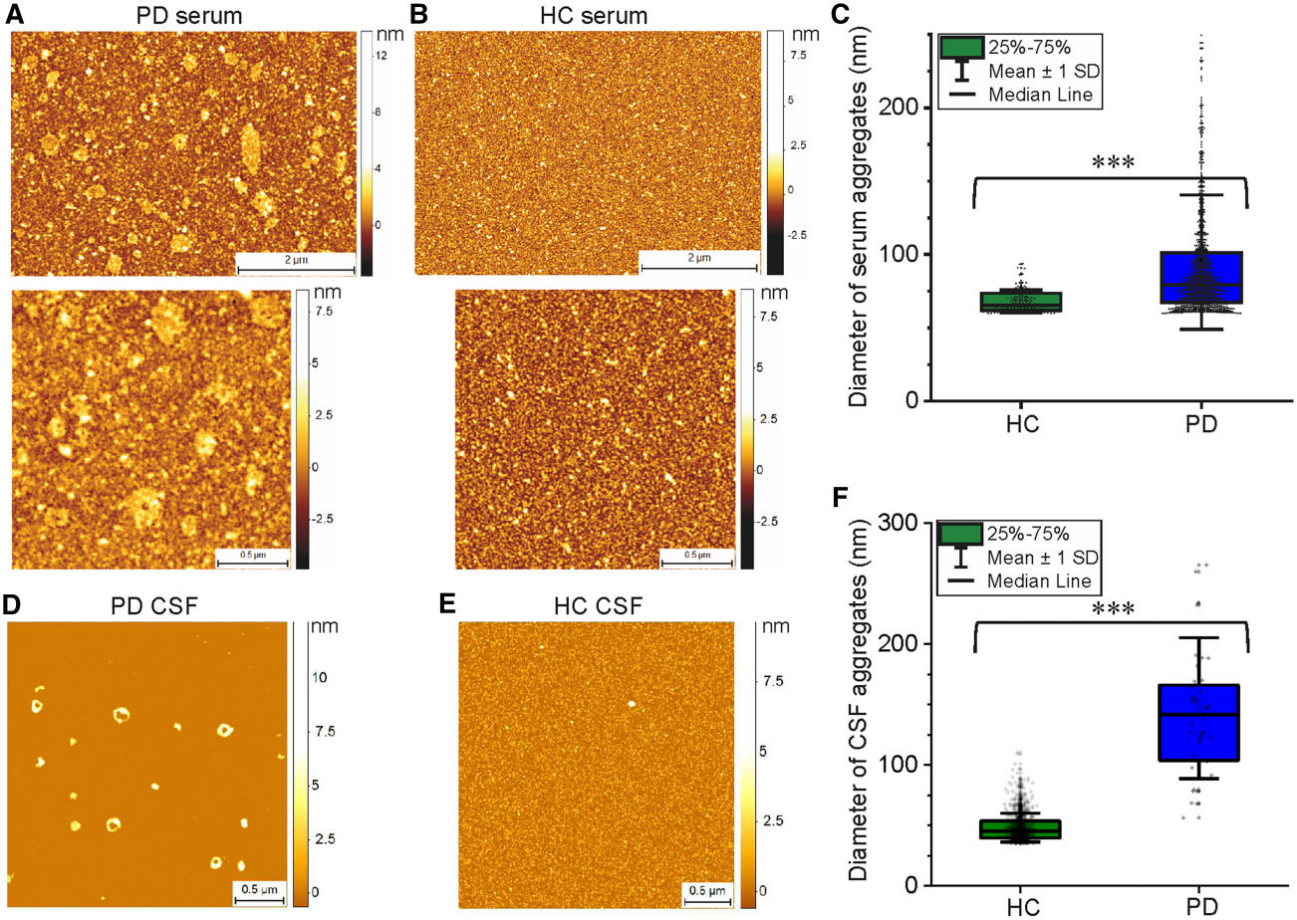
**Characterization of the protein aggregates detected in Parkinson’s disease serum and CSF samples compared to controls using AFM.** Representative AFM images of a Parkinson’s disease (PD, **A**) compared to healthy control (HC, **B**) serum sample and a Parkinson’s disease (**D**) versus control (**E**) CSF dried onto mica. Diameter of aggregates (mean ± SD) detected in these serum and CSF samples are shown in **C** and **F**, respectively.

We then confirmed the results of the AD-PAINT analysis in serum and CSF samples by performing phase-controlled and high resolution AFM imaging of the 3D morphology of the aggregates^19,20^ (Fig. 5). A single molecule statistical analysis of the aggregates enabled us to observe significantly larger aggregates in Parkinson’s disease compared to control groups for both serum and CSF, with the Parkinson’s disease samples having a wider range of diameters, which is in good agreement with the AD-PAINT measurements (Fig. 4). Additional AFM images with the similar morphology of serum aggregates for Parkinson’s disease and control cases from another replicate are shown in Supplementary Fig. 3.

**Figure 6:**
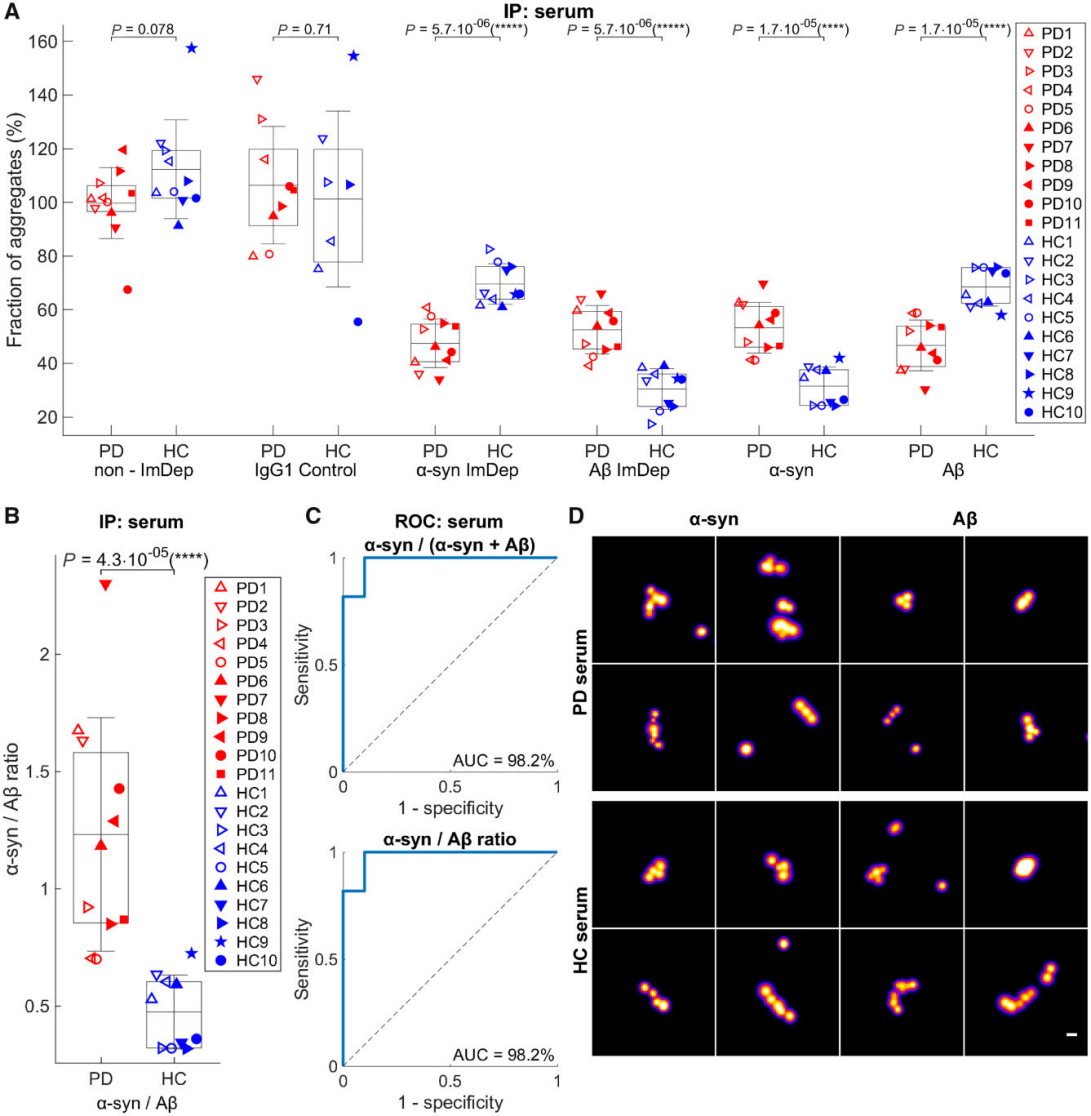
**Aggregates with pathogenic β-sheet structure detected using AD-PAINT following immunodepletion of α-syn and amyloid-β protein aggregates in Parkinson’s disease (*n* = 11) and healthy control (*n* = 10) serum samples from two independent cohorts pooled together.** (**A**) Quantification of the relative content (%) of β-sheet α-syn and amyloid-β aggregates in Parkinson’s disease (PD) versus healthy control (HC) serum samples. The percentage of α-syn or amyloid-β aggregates in each sample was determined as the difference in the number of detected aggregates between the neat and α-syn or amyloid-β immunodepleted serum normalized to their sum (α-syn + amyloid-β). In turn, the data for neat, IgG1 control, α-syn and amyloid-β immunodepleted serum of each subject were normalized to the sum of the aggregate number in α-syn and amyloid-β immunodepleted samples (α-syn + amyloid-β ImDep). (**B**) Quantification of the β-sheet α-syn/amyloid-β ratio retrieved from **A** for the same serum samples. In **A** and **B**, different subjects are indicated by a specific empty symbol for the first independent cohort and a specific filled symbol for the second cohort of samples (legend). The data are shown as mean ± SD, and the lower and upper boundaries of the box indicate the 25th and 75th percentiles, respectively. The statistical significance for the difference in the serum aggregate composition between Parkinson’s disease and healthy control groups was established by the permutation (exact) test. *P < 0.05, **P < 0.01. (**C**) ROC analysis for disease status classification by relative β-sheet α-syn content (optimal threshold = 0.41) as well as the β-sheet α-syn/amyloid-β ratio (optimal threshold = 0.7) showing high performance of each tested biomarker (AUC = 98.2% for each). (**D**) Examples of super-resolved α-syn and amyloid-β aggregates present in Parkinson’s disease and healthy control serum. Scale bar = 100 nm.

Finally, we performed additional experiments to better characterize the aggregates present. Since the AD-PAINT aptamer detects both α-syn and amyloid-β aggregates with β-sheet structure, we performed α-syn and amyloid-β immunodepletion experiments (see a schematic of experimental design in Fig. 2). As a selectivity and specificity control, we first confirmed that the immunodepletion significantly removes the target aggregates without depleting other aggregates using synthetic aggregates of α-syn and amyloid-β individually at 500 nM as well as their mixture at 500 nM and 2 μM concentrations of both aggregates (Supplementary Fig. 7). As the level of aggregates was too low in the CSF to do the immunodepletion (∼20-fold less than in serum), we performed experiments only on serum from two independent cohorts of five Parkinson’s disease patients and five age-matched control subjects (Cohort 1, Table 2), and six Parkinson’s disease and five control subjects (Cohort 2, Table 2), comparing the levels of the super-resolved α-syn individual deposits before and after immunodepletion using AD-PAINT. In the first cohort the samples were undiluted (neat), whereas in the second cohort the serum was significantly more concentrated, possibly due to a significantly shorter storage duration of less than 6 months at −80°C, resulting in too high a density of aggregates for super-resolution imaging. The serum from the second cohort was therefore diluted 10-fold. In both cohorts, this experiment confirmed that the proportion of α-syn and amyloid-β aggregates to the total β-sheet aggregate content was significantly different for Parkinson’s disease and control serum with an increased β-sheet α-syn/amyloid-β ratio in the Parkinson’s disease group (Supplementary Figs 4 and 5) and that this ratio was found to be invariant regardless of the aggregate concentration in the sample (dilution used). Therefore, the samples in these two cohorts were combined. In the combined samples, the difference in the β-sheet α-syn/amyloid-β ratio between Parkinson’s disease and control samples became more significant (*P* = 4.3 × 10^−5^; Fig. 6B). Parkinson’s disease serum aggregates were found to consist of, on average, ∼50% α-syn and 50% amyloid-β, whereas healthy control serum contained ∼30% α-syn and 70% amyloid-β (Fig. 6A). Importantly, the absolute levels of β-sheet α-syn and amyloid-β aggregates in Parkinson’s disease and control serum were overlapping in Parkinson’s disease patients and control subjects, while the β-sheet α-syn/amyloid-β ratios were separated between Parkinson’s disease and control samples in all investigated cohorts (see Supplementary Figs 4C, 5C and 6A for the first, second and combined cohorts). To assess whether our novel aggregate measurements constitute a useful biomarker, we performed ROC analysis which indicated that the β-sheet α-syn/amyloid-β ratio (optimal threshold = 0.7), as well as the relative α-syn levels of serum aggregates alone (optimal threshold = 0.41), distinguish Parkinson’s disease patients from age-matched controls with accuracy of 98.2% (AUC = 98.2%, optimal sensitivity = 100% and optimal specificity = 90% for each tested biomarker) (Fig. 6C). ROC analysis gave AUC of 100% in both independent Cohorts 1 and 2 (Supplementary Figs 4D and 5D). In all cohorts, we found no correlations between these biomarkers and clinical measures of Parkinson’s disease, but the sample size was too small to adequately evaluate this. We also compared the size distributions of α-syn and amyloid-β aggregates between Parkinson’s disease and healthy controls and found no significant difference in the length of amyloid-β aggregates, but α-syn aggregates were longer in the Parkinson’s disease samples (Supplementary Fig. 6B and E). Representative images of individual super-resolved α-syn and amyloid-β aggregates detected with AD-PAINT in Parkinson’s disease and healthy control serum are shown in Fig. 6D.

## Discussion

Previous experiments using ELISA-based methods have detected an increase in the amount of oligomeric α-syn in Parkinson’s disease compared to control human CSF^24–27^ by a factor of ∼2, but no differences in serum.^28^ Whilst this approach may allow for specific detection of oligomeric as opposed to monomeric α-syn, it provides no information about the number and size distribution of the aggregates present. In this work, we performed a pilot study using three methods to quantify and characterize single aggregates. First, we used a confocal method to detect PFTAA-active aggregates with a β-sheet-rich secondary structure. Our results revealed no significant difference in the number of aggregates between Parkinson’s disease and control groups in both serum and CSF. We then used an aptamer that binds to fibrillar aggregates of α-syn and amyloid-β, and again found a comparable number of aggregates in Parkinson’s disease compared to control serum as well as CSF. The aggregates that were detected ranged in size from 20 to 200 nm, in both serum and CSF, with a significantly higher proportion of aggregates larger than 150 nm in Parkinson’s disease versus control serum. The presence of these larger aggregates in Parkinson’s disease serum was also confirmed by direct imaging of aggregate morphology using high resolution AFM.

The reported increase in levels of oligomeric α-syn in Parkinson’s disease compared to control CSF in previous ELISA experiments^24–27^ could be due to more aggregates being present or the same number of aggregates being present but these aggregates being larger. Our data show a small increase in the proportion of larger aggregates but this does not explain the more pronounced increase in oligomeric α-syn observed with ELISA. However, an important difference in our AD-PAINT method is in its capability to simultaneously detect both α-syn and amyloid-β aggregates with high β-sheet content, hence the total number of aggregates observed represents a combination of these different chemical species, which could be present in different proportions. Although the aggregate levels were too low to allow us to resolve this question in the CSF samples, we were able to investigate the relative proportions of α-syn and amyloid-β in serum samples by performing AD-PAINT following immunodepletion of these proteins from serum. Our results reveal that the ratio of α-syn to amyloid-β is significantly increased in Parkinson’s disease compared to control serum, while the total number of aggregates in Parkinson’s disease and control groups appears comparable. In contrast, the absolute number of α-syn and amyloid-β aggregates varied for each individual independent of the disease status (Parkinson’s disease or control) leading to the overlap of their levels between Parkinson’s disease and control serum. This finding implies that the absolute levels of protein aggregates may not be a useful biomarker in Parkinson’s disease but, in contrast, the ratio of aggregates of different proteins is a promising candidate biomarker. Furthermore, the utility of the α-syn to amyloid-β ratio as a biomarker was strengthened by our finding that this ratio discriminated Parkinson’s disease from control serum in two independent cohorts, despite the fact that serum storage time and total aggregate concentration differed in the two cohorts. However, since we have observed an effect of storage time on absolute aggregate concentration in our serum samples, it is important that future studies should carefully control for this parameter and take it into consideration when using the α-syn to amyloid-β ratio as a biomarker.

Our measurements of aggregate size distribution showed that a higher proportion of larger aggregates were present in serum versus CSF. Interestingly, our PFTAA experiments also indicated that aggregates with higher intermolecular β-sheet content were 20-fold more concentrated in serum than CSF. This is a solution-based method and detects all aggregates that flow through the confocal volume and hence provides a reliable estimate of the relative concentrations. Given that one would expect a significant dilution when the aggregates from the CSF enter the blood, this finding suggests that a high proportion of serum aggregates might be of peripheral origin. α-Syn aggregates are found in the gut early in the course of Parkinson’s disease and may propagate via the vagus nerve to the brainstem.^29^ α-Syn aggregates are also detectable in erythrocytes at higher levels in Parkinson’s disease versus controls.^30^ It is also possible that the aggregates secreted from the brain via the CSF may accumulate in the blood, due to slow removal, particularly the larger and more mature fibrillar aggregates, which is in line with our experiments. Irrespective of their peripheral or central origin, our data highlight that detection of aggregates in serum may be a useful biomarker in Parkinson’s disease.

Our data show that there are detectable aggregates of both α-syn and amyloid-β present in both the CSF and serum in Parkinson’s disease patients and healthy control subjects, consistent with the concept that secretion is a major route of aggregate removal by cells under normal conditions, and that these aggregates are altered in size and in their relative proportions in the early stages of Parkinson’s disease. The fact that we observed a small fraction of large aggregates over 100 nm in both control and Parkinson’s disease CSF and serum suggests that some aggregates spend sufficient time in the cell to grow to this size before secretion, as the concentration of α-syn and amyloid-β is too low in the CSF or serum for growth to continue after the aggregates are secreted. Any cellular stress due, for instance, to inflammation or mitochondrial dysfunction, might reduce the cell’s secretion capacity resulting in aggregates growing larger before secretion and possibly also leading to accumulation of aggregates in neurons. Disruption of protein homeostasis in Parkinson’s disease appears to preferentially lead to small increases in the number and size of secreted aggregates of α-syn, compared to aggregates of amyloid-β. It is also possible that small increases in the α-syn aggregates over a prolonged time period may play a role in driving the disease. Importantly, we have observed changes in both α-syn and amyloid-β aggregates, reinforcing the idea that co-pathologies are involved in Parkinson’s disease, and highlighting the importance of measuring both these species.

We have also observed a negative correlation in total number of aggregates between matched serum and CSF samples in healthy controls, but a lack of correlation between CSF and serum aggregates in Parkinson’s disease. One possible explanation for the observed serum versus CSF correlation in healthy serum is that aggregates in controls are mainly generated in the brain, and are cleared from CSF into the blood at different rates of efficiency (so levels remain high in the CSF and low in the serum in some individuals but are cleared from CSF and hence become high in the serum in other individuals). In contrast, in patients with Parkinson’s disease, it is likely that aggregates are generated both in the periphery and in the brain, hence the lack of correlation between CSF and serum aggregates. A larger study would be needed to confirm this.

In conclusion, we found that fibrillar serum aggregates range in size from 20 to 200 nm with an increased number and proportion of larger aggregates (longer than ∼150 nm) in the serum in early-stage Parkinson’s disease patients compared to control subjects. This may reflect accumulation of brain-derived aggregates or peripherally-generated aggregates in the blood over time. These serum aggregates consist of both α-syn and amyloid-β species with a significantly higher proportion of α-syn aggregates being found in Parkinson’s disease patients compared to control subjects. The serum α-syn/amyloid-β ratio measured using our single-aggregate method has high diagnostic potential with accuracy of almost 100% (AUC = 98.2%), albeit in a relatively small group of 11 Parkinson’s disease patients and 10 control subjects. Nonetheless, our results identify a new potential biomarker for Parkinson’s disease diagnosis. We now plan to simplify our methodology, which is currently quite time consuming, prior to validation work in a larger cohort of samples to confirm Parkinson’s disease versus control differences, and to investigate longitudinal changes in Parkinson’s disease patients over time to assess whether detection of α-syn and amyloid-β aggregates is a useful strategy for monitoring Parkinson’s disease progression and the effectiveness of therapies.

## Supplementary Material

awab306_Supplementary_Data

## Abbreviations

ACE-III: Addenbrooke’s Cognitive Examination version 3
α-syn: α-synuclein
AD-PAINT: Aptamer DNA-PAINT
AUC: area under the curve
AFM: atomic force microscopy
MDS-UPDRS: Movement Disorder Society Unified Parkinson’s Disease Rating Scale
ROC: receiver operating characteristic
