# Corrigendum to: Imaging protein aggregates in the serum and cerebrospinal fluid in Parkinson’s disease

**DOI:** 10.1093/brain/awab346

**Published:** 2021-09-26

Evgeniia Lobanova, Daniel Whiten, Francesco S. Ruggeri, Christopher G. Taylor, Antonina Kouli, Zengjie Xia, Derya Emin, Yu P. Zhang, Jeff Y. L. Lam, Caroline H. Williams-Gray, David Klenerman. Imaging protein aggregates in the serum and cerebrospinal fluid in Parkinson’s disease. *Brain*. 2021;awab306. doi:10.1093/brain/awab306

The authors apologize for misspelling the name of Christopher G. Taylor. This has been corrected.

