# Supplementary material for: Imaging protein aggregates in the serum and cerebrospinal fluid in Parkinson’s disease
Source: Brain. 2021 Aug 19;145(2):632–43. doi: 10.1093/brain/awab306 (PMC9014748; doi:10.1093/brain/awab306)
Supplement: awab306_Supplementary_Data [file awab306_supplementary_data.pdf]

Supplementary material for “Imaging protein aggregates in the serum and cerebrospinal fluid in Parkinson’s disease”

Evgeniia Lobanova[1], Daniel Whiten[1], Francesco S. Ruggeri[1,2,3], Chris Taylor[1], Antonina Kouli[4], Zengjie Xia[1], Derya Emin[1,5], Yu P. Zhang[1], Jeff Y.L. Lam[1], Caroline H. Williams-Gray[4†#] and David Klenerman[1,5†#]

[†]Joint senior authors

[1]Department of Chemistry, University of Cambridge, Lensfield Road, Cambridge CB2 1EW, United Kingdom

[2]Laboratory of Organic Chemistry, Stippeneng 4, 6703 WE, Wageningen University, the Netherlands

[3]Laboratory of Physical Chemistry, Stippeneng 4, 6703 WE, Wageningen University, the Netherlands

[4]Department of Clinical Neurosciences, University of Cambridge, Cambridge CB2 0PY, United Kingdom

[5]UK Dementia Research Institute at Cambridge, Cambridge CB2 0XY, United Kingdom

[#]Correspondence to: chm27@cam.ac.uk or dk10012@cam.ac.uk

The supplementary material contains the additional results on the confocal imaging (Fig. 1), AD-Paint (Fig. 2), AFM (Fig.3) and immunodepletion (Figs. 4-7) experiments as presented in our main manuscript.

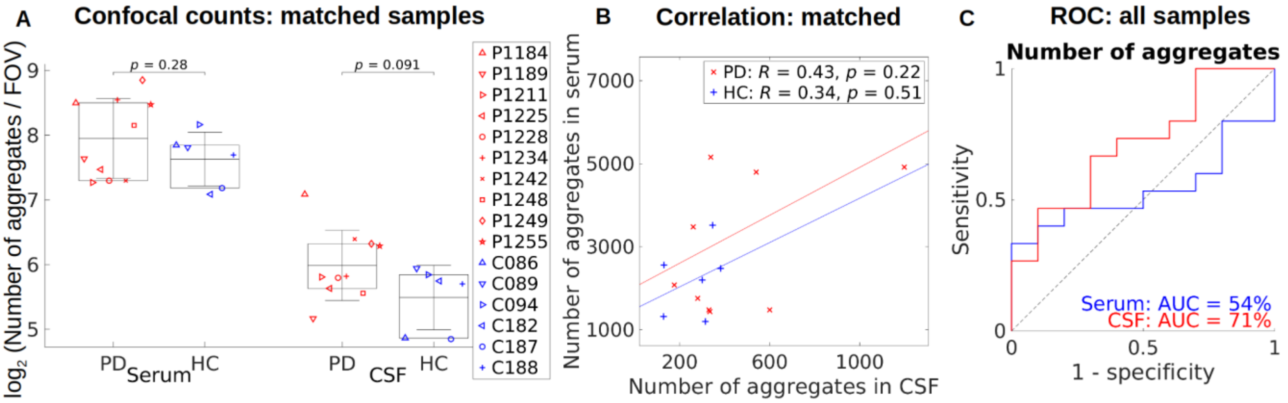

Fig. 1. (**A**) Confocal analysis of the aggregates present in matched PD CSF and serum (n = 10) compared to controls (n = 6). Serum was diluted by a factor of 40 and CSF by a factor of 2. Data are shown as mean ± SD with each circle representing individual patients and plotted in log_2 scale. The lower and upper boundaries of the box indicate the 25th and 75th percentiles, respectively. PD versus HC comparisons using the permutation (exact) test were insignificant ($p > 0.05$). (**B**) Correlation between the number of aggregates in serum versus CSF for matched PD (red crosses) and HC (blue pluses) samples. Pearson’s correlation coefficients (R) and

p-values are indicated on the figure. (**C**) ROC analysis for the disease status classification by the number of aggregates detected in serum and CSF for all samples (n = 15 PD and 10 HC).

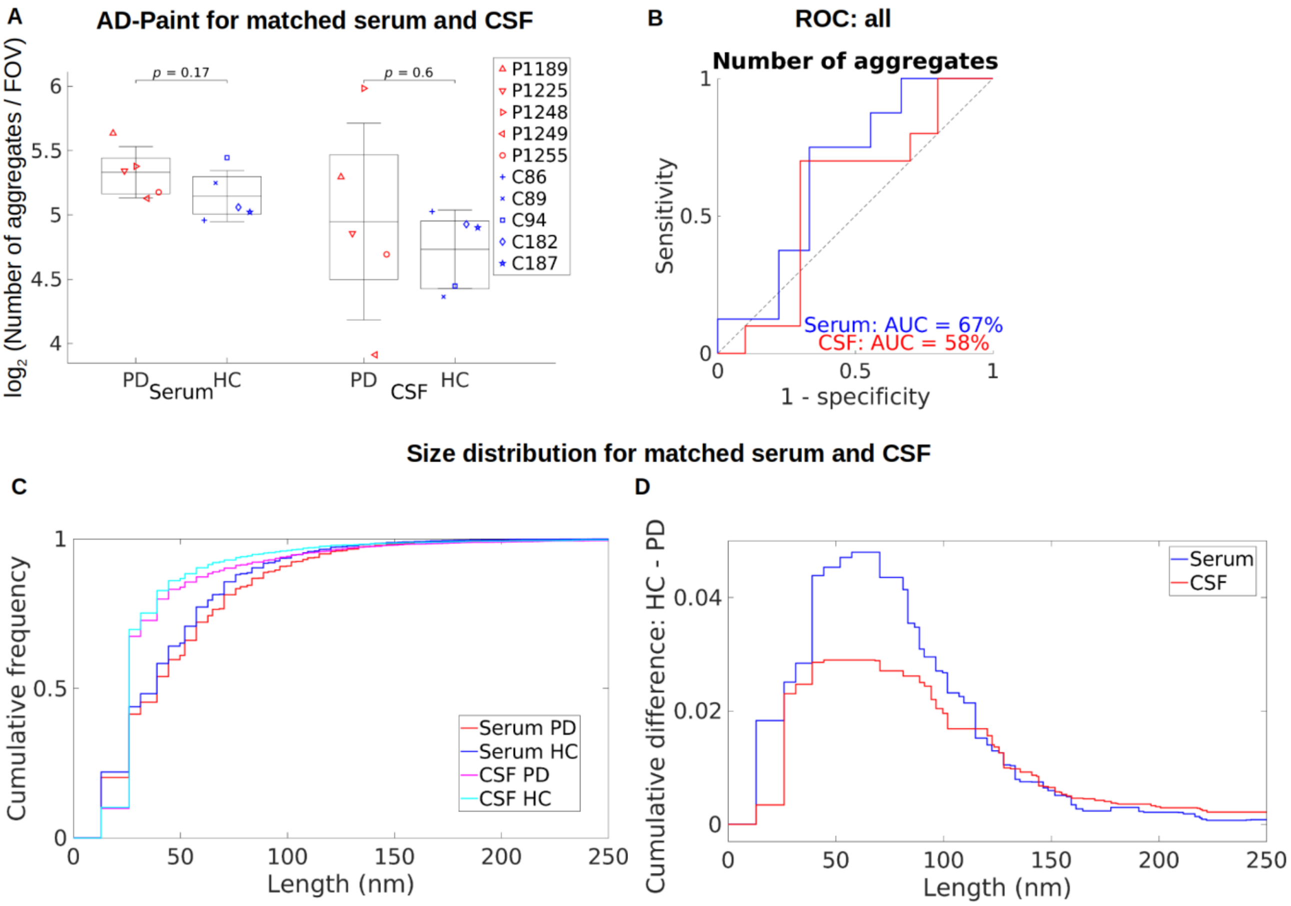

Fig. 2. (**A**) Quantification of the super-resolved aggregates detected in matched CSF and serum samples from PD cases (n = 5) compared to controls (n = 5), using AD-PAINT. Serum aggregates are shown undiluted and CSF was diluted 2-fold. Data are shown as mean ± SD with each circle representing individual patients and plotted in $\log_2$ scale. The lower and upper boundaries of the box indicate the 25th and 75th percentiles, respectively. The permutation (exact) test, *p* values were insignificant ($p > 0.05$). (**B**) ROC analysis for PD versus control classification by the number of super-resolved aggregates detected in serum and CSF for all samples (n = 8 PD and 9 HC serum, n = 10 PD and 10 HC CSF). (**C**) Cumulative length distributions for the matched PD and control serum and CSF samples measured by AD-Paint. (**D**) Difference between PD and control cumulative length distributions for matched CSF and serum retrieved from (C).

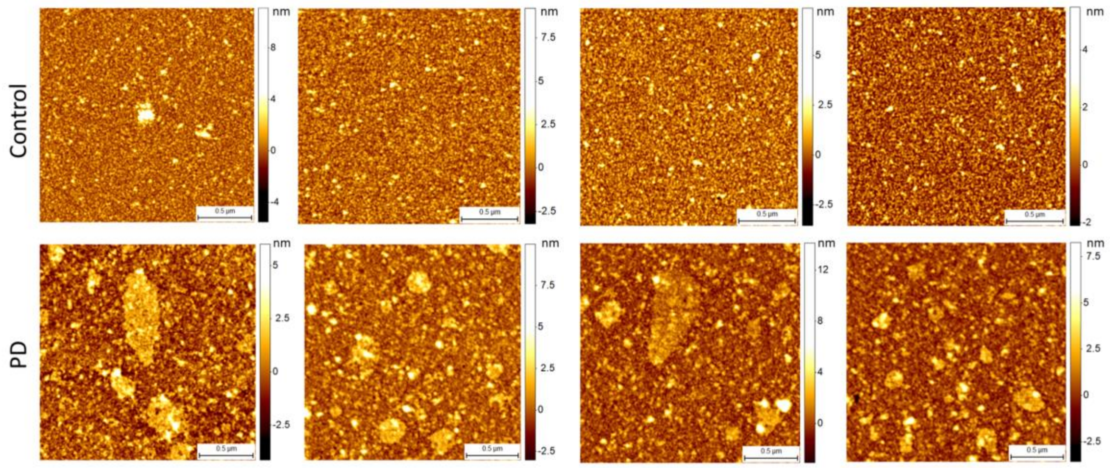

Fig. 3. AFM images of 1 PD compared to 1 control serum sample from another replicative experiment.

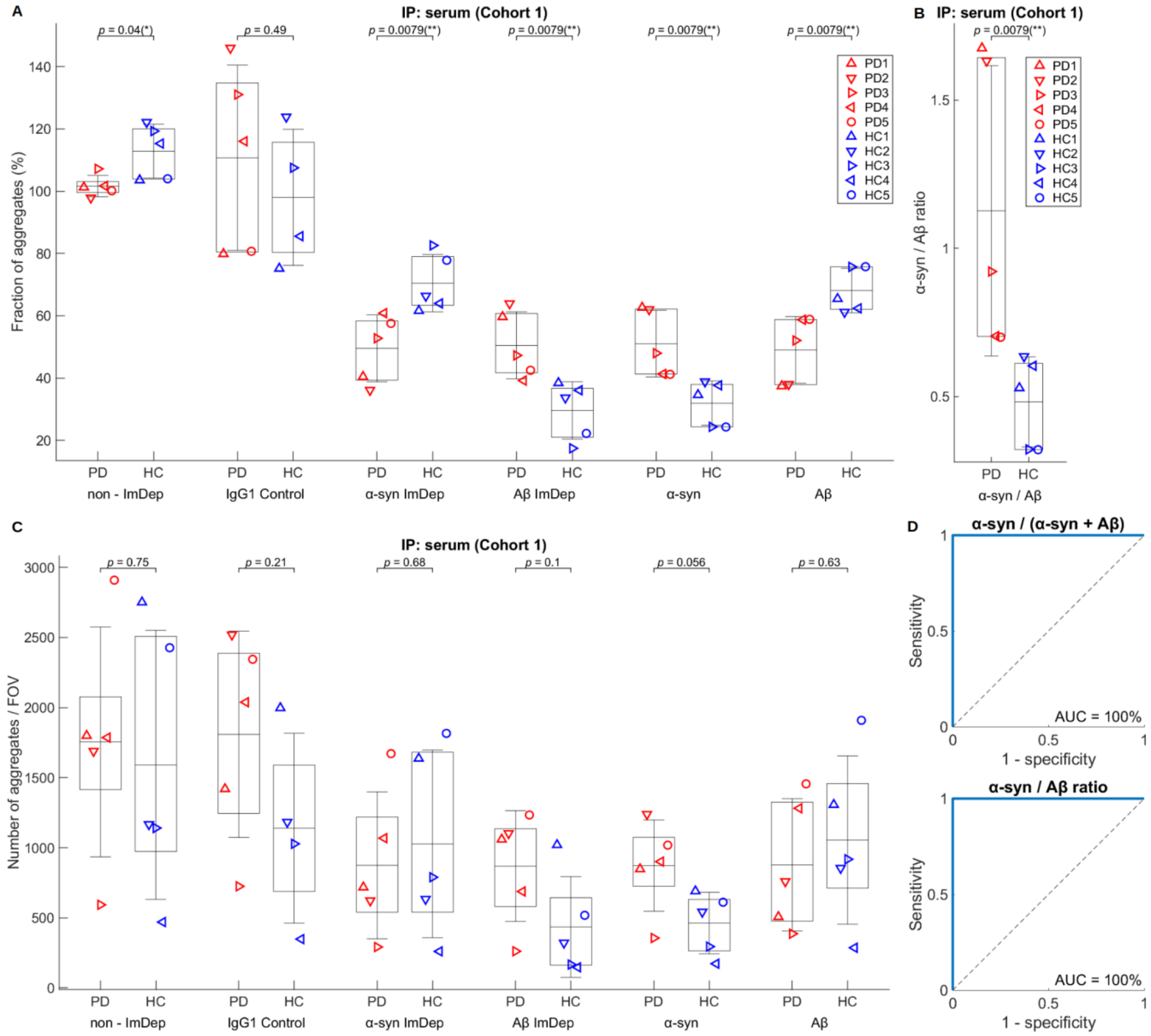

Fig. 4. Quantification of the (**A**) relative content (%) and the absolute number (**C**) of β-sheet α-syn and Aβ aggregates in Cohort 1 (n = 5 PD vs 5 HC serum) found using AD-PAINT of the serum before and after

immunodepletion (ImDep). Serum was undiluted (neat). (**B**) Quantification of the β-sheet α-syn/Aβ ratio retrieved from (A) for the same serum samples. The number of α-syn or Aβ aggregates in each sample was determined as the difference in the number of detected aggregates between the neat and α-syn or Aβ ImDep serum. The data are shown as mean ± SD, and the lower and upper boundaries of the box indicate the 25th and 75th percentiles, respectively. The statistical significance for the difference in the serum aggregate composition between PD and HC groups was established by the permutation (exact) test when *p* values below 0.05. (**D**) ROC analysis for disease status classification by relative β-sheet α-syn content as well as the β-sheet α-syn/Aβ ratio showing high performance of each tested biomarker (AUC = 100% for each).

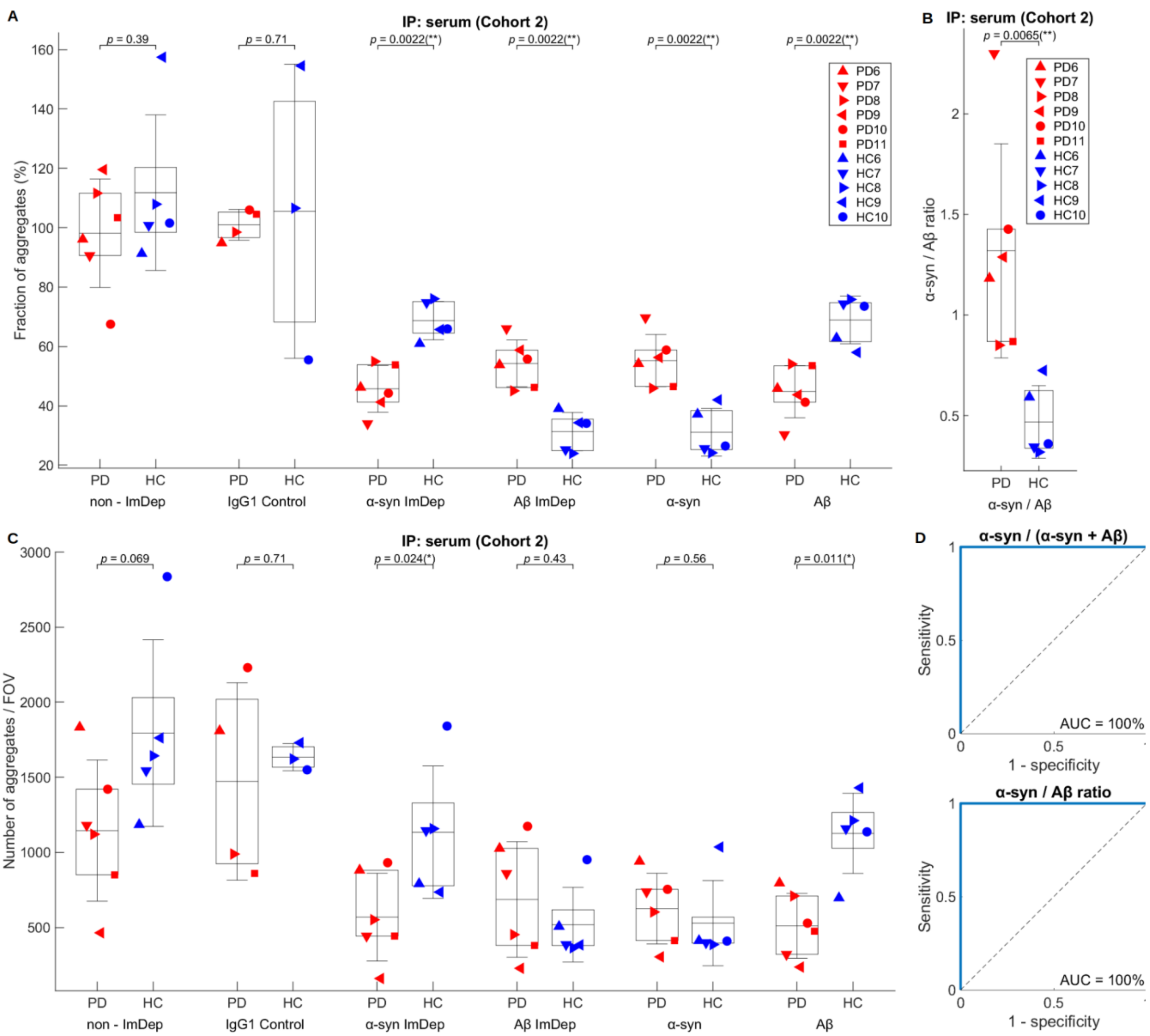

Fig. 5. Similar data as shown in Fig.5 but for cohort 2 (n = 6 PD and 5 HC serum). 10-fold dilution was used for serum AD-PAINT imaging.

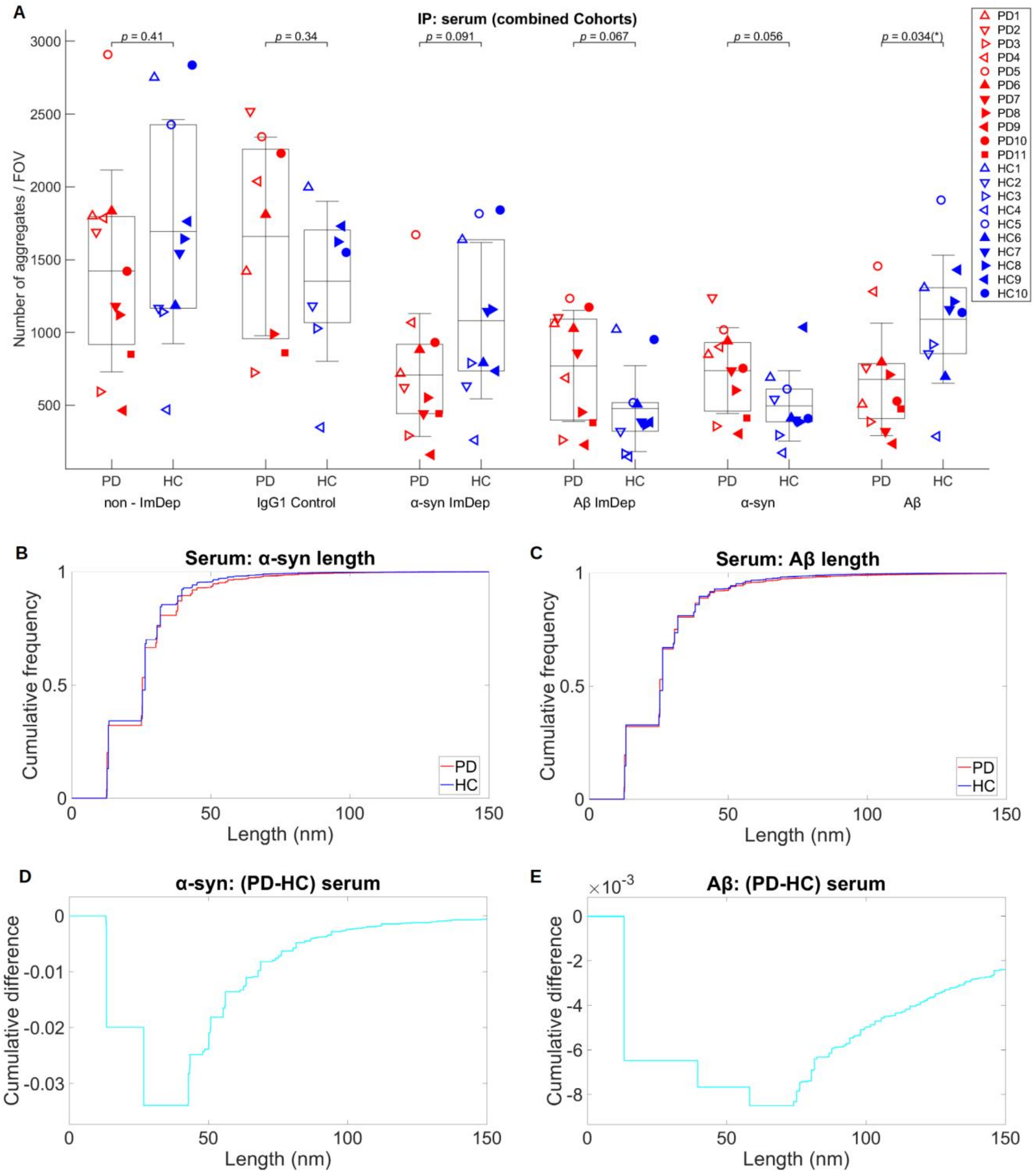

Fig. 6. (**A**) Quantification of the number of β-sheet α-syn and Aβ aggregates in the combined serum cohort (n = 11 PD vs 10 HCs) found using AD-PAINT of the serum before and after immunodepletion (ImDep). (**B-C**) Cumulative length distributions of α-syn (B) and Aβ (C) aggregates for the PD and control serum by AD-PAINT. (**D-E**) Difference between PD and control cumulative length distributions for α-syn (D) and Aβ (E) retrieved from (B, C).

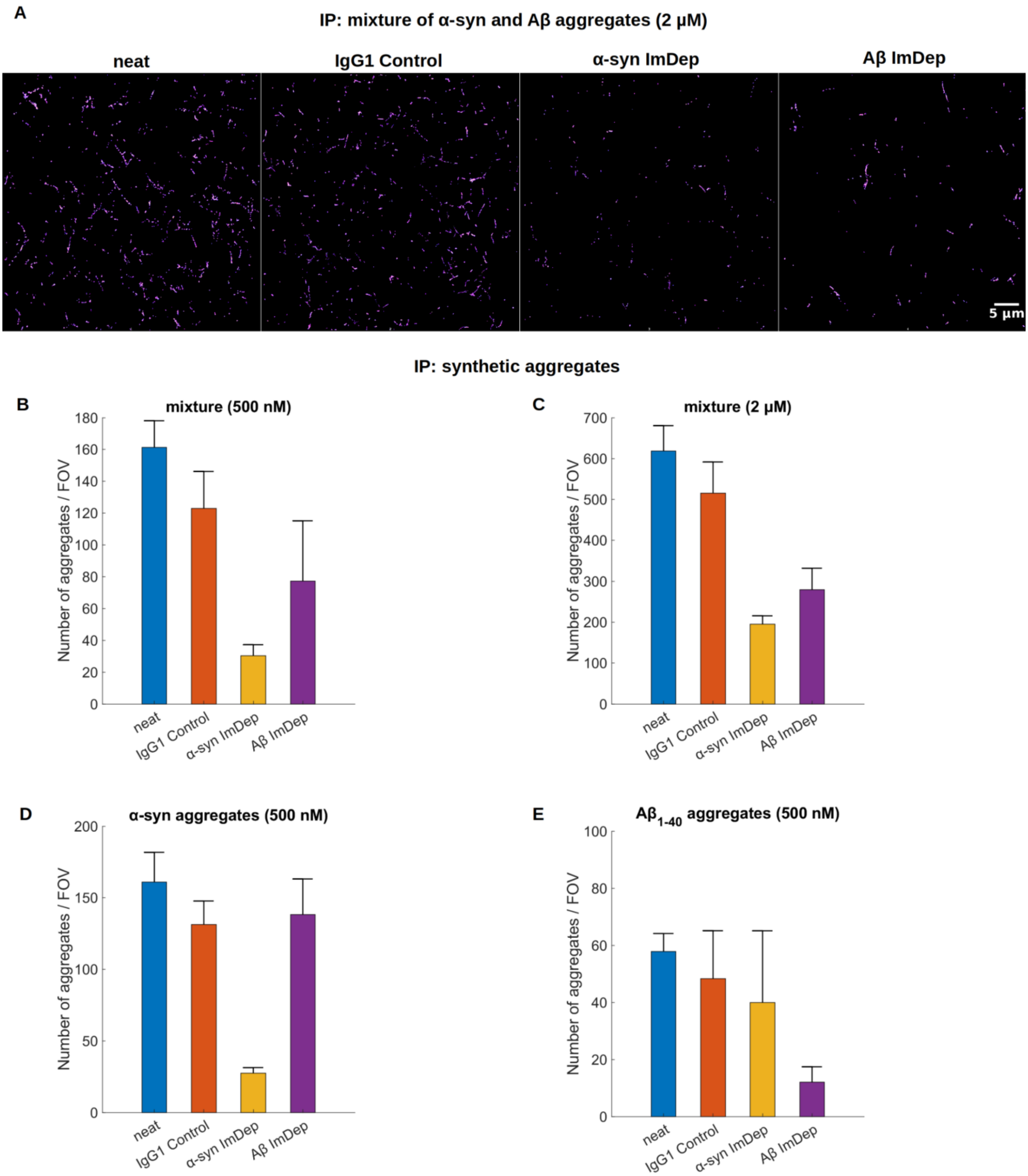

Fig. 7. Selectivity and specificity control experiments of the number of α-syn and Aβ aggregates detected with AD-PAINT before and after immunodepletion of (**B**, **C**) a mixture of synthetic α-syn and Aβ$_{1-40}$ (500 nM and 2 μM, respectively), (**D**) α-syn (500 nM) and (**E**) Aβ$_{1-40}$ (500 nM) aggregates with the target antibody protein (selectivity control) compared non-target ones (specificity control). The data are shown as mean ± SEM. (**A**) Super-resolution AD-PAINT images for α-syn, Aβ and non-target IgG control immunodepleted mixture (2 μM) compared to un-depleted neat.
